# Comparative analysis of positioning accuracy of Samsung Galaxy smartphones in stationary measurements

**DOI:** 10.1371/journal.pone.0215562

**Published:** 2019-04-18

**Authors:** Tomasz Szot, Cezary Specht, Mariusz Specht, Pawel S. Dabrowski

**Affiliations:** 1 Department of Natural Sciences, Gdansk University of Physical Education and Sport, Gdansk, Poland; 2 Department of Geodesy and Oceanography, Gdynia Maritime University, Gdynia, Poland; 3 Department of Transport and Logistics, Gdynia Maritime University, Gdynia, Poland; Northwestern University, UNITED STATES

## Abstract

Achieving single meter positioning accuracy by portable mobile devices still poses a major challenge to the satellite signal receivers constructors, despite gradual constellation completing process and the progress achieved in last decades. Nowadays popular smartphones are multifunctional devices that serve also as a personal navigation tool in navigation and sport activities using the global navigation satellite systems (GNSS) receivers installed. It would seem that introducing newer models to the global market would cause constant progress in the accuracies obtained, however, the study results do not confirm that. This study focused on Galaxy series smartphones of Samsung, one of the leading manufacturers worldwide, to examine its technological progress. The aim was to verify the thesis using statistical models and analyses to compare succeeding generations of smartphones on six devices from the series. The authors conducted two synchronous stationary measurement campaigns of 24 and 12 hours with one-second interval in obstacle-free environment which provided 70000+ and 30000+ statistical samples of position measurements. The reference values of true smartphones coordinates were determined by means of state-of-the-art precise surveying instruments and geodetic calculations. The results indicate that two newest generations of the Galaxy series included in the research, namely S6 and S7, obtained lower accuracies than their predecessors. Against the backdrop of lack of public availability of smartphones technical parameters, the conducted research results are relevant especially to smartphones positioning service users community.

## Introduction

Smartphones have become an inherent element of daily life of developed societies in the past decade. With comprehensive accessories, readily installable applications connected to the internet, they have in fact become upgraded multifunctional devices that help process, store and disseminate various types of information, regardless of location and time. Their increasing capacities have made them the number one worldwide sale in 2013, as compared to other mobile phones [1]. According to recent global studies, they are owned by 72% of adults in the United States, 67% in Canada, 77% in Australia [2], and the estimated total number of users will reach nearly 2.9 billion in 2020 [3]. The leading global vendors are Samsung and Apple, with approximately 18% of the global market each [4].

**Fig 1:**
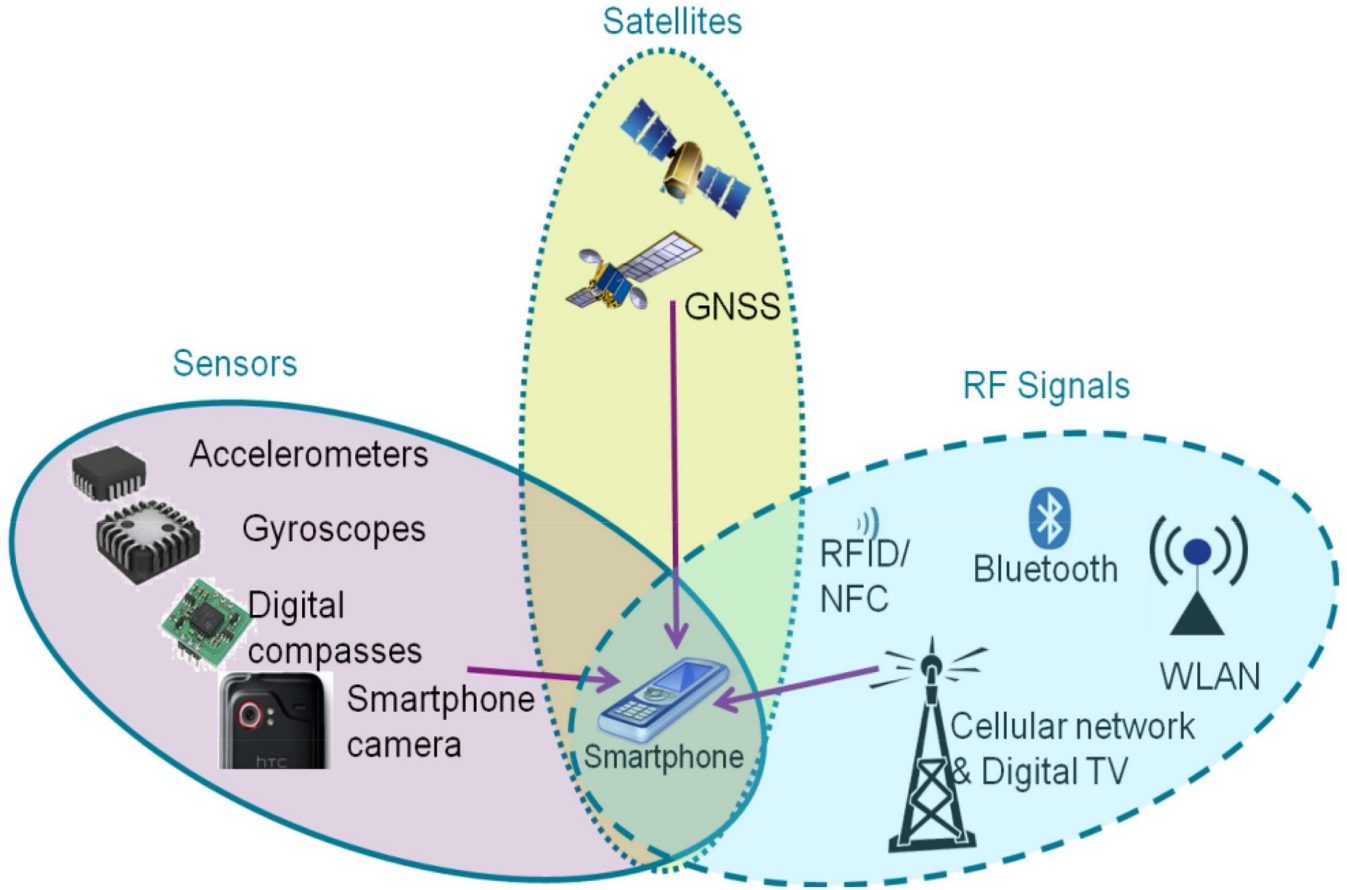
Three families of smartphone-based positioning solutions [13].

In addition to basic features typical for most mobile phones, smartphones come with a range of additional systems and sensors, including multi-axis accelerometers and gyroscopes, magnetometers, cameras, Global Navigation Satellite Systems (GNSS) receivers, light intensity sensors, and fingerprint readers or iris scanners in some cases. The use of these elements coupled with the ingenuity of mobile application developers has resulted in thousands of free or paid applications available to users. As the current leading operating system is Android (85% market share), most applications and devices are developed for this platform [5]. It is noteworthy that a smartphone, as a versatile device, can replace many other dedicated devices, such as car and tourist navigation tools or cameras. A specific group of smartphone applications deals with positioning (Fig 1). Advanced motion, location and navigation applications that combine different positioning solutions can, for example, recognise the human body condition [6], the type of activity [7–9] or perform measurements of physical activity and influence it [10–11]. They can also be used in intelligent transport systems [12].

A very large group of applications and solutions is based entirely or in part on global and local satellite navigation systems [14–18]. The latest smartphones, such as Samsung Galaxy (models: S5-S8), receive signals of not only fully operational systems Global Positioning System (GPS) (United States) and GLONASS (Russia), but also of those being still developed—such as Chinese BeiDou and European Galileo. Using more than one system speeds up the positioning process and increases the accuracy of position determination [19–20]. This is important because potential failure in high accuracy achievement can lead to errors, for instance in navigating a moving vehicle, determining the route or speed of a person engaged in physical, tourist or other activities in the Location Based Services (LBS) [21]. Mobile phone manufacturers do not usually inform users about technical aspects of implemented GNSS receivers. It is known, however, that they belong to a group of autonomous receivers determining distances based on correlation properties of pseudo-random codes—in the same way as popular receivers dedicated to sport and recreation.

The concept of position determination accuracy (positioning) is a key parameter defining GNSS receivers. The GPS system user segment includes both precise geodetic receivers used in the highest accuracy applications (e.g. Earth crust displacements monitoring) and lower accuracy receivers that determine the position for recreational and tourist purposes.

In geodesy and navigation, positioning accuracy using GNSS satellite navigation systems is carried out via [22–23]:

- static (stationary) measurements, where the position coordinates obtained from a receiver measurements are referenced to known reference coordinates—geodetic coordinates (predictable accuracy) or to their position averaged in a session (repeatable accuracy);
- kinematic (in motion) measurements, where position coordinates obtained from measurements are referenced to a predetermined track.

In summary, the purpose of this article is to provide a comparative analysis of the positioning accuracy of Samsung Galaxy smartphones, one of the world's leading manufacturers of mobile phones using static (stationary) measurements.

## Materials and methods

### Subject of study

For the comparative analysis five Samsung Galaxy S series smartphones were chosen: 3 Mini, 4, 5, 6, 7, and Galaxy Y. Selected technical parameters are presented in Table 1. Originally, the authors intended to use first generation Galaxy S and Mini 2 phones, however, the devices were discarded due to uncontrolled application downtime. The smartphone accuracy analyses carried out so far have commonly adopted the methodology of using one device in a research [24–25]. This would confirm the thesis that individual devices released on the market are identical in terms of technical parameters. Therefore, quality tests (both in terms of firmware and hardware) confirm the repeatability of individual devices. A more detailed analysis of GNSS antenna parameters used in smartphones was carried out in [26]. In addition, it should be noted that research [27–29] that focused on the precise Real Time Kinematic measurement technology (providing centimetre accuracy) also used single copies of mobile devices. Therefore, since even if analyses of such high accuracy are carried out with the use of a single model, such approach is even more legitimate for much less accurate position estimation by smartphones GNSS receivers.

**Table 1:** Selected parameters of analysed Samsung smartphones.

Samsung	Samsung Galaxy Series					
	Y	S3 Mini	S4	S5	S6	S7
**Model number**	GT-S5360	GT-I8190N	GT-I9505	SM-G900F	SM-G920F	SM-G930
**Market launch**	2011	2012	2013	2014	2015	2016
**GPS**	+	+	+	+	+	+
**GLONASS**	-	+	+	+	+	+
**BeiDou**	-	-	-	+	+	+
**RAM**	384 MB	1.5 GB	2 GB	2 GB	3 GB	4 GB

### Measurements

**Fig 2:**
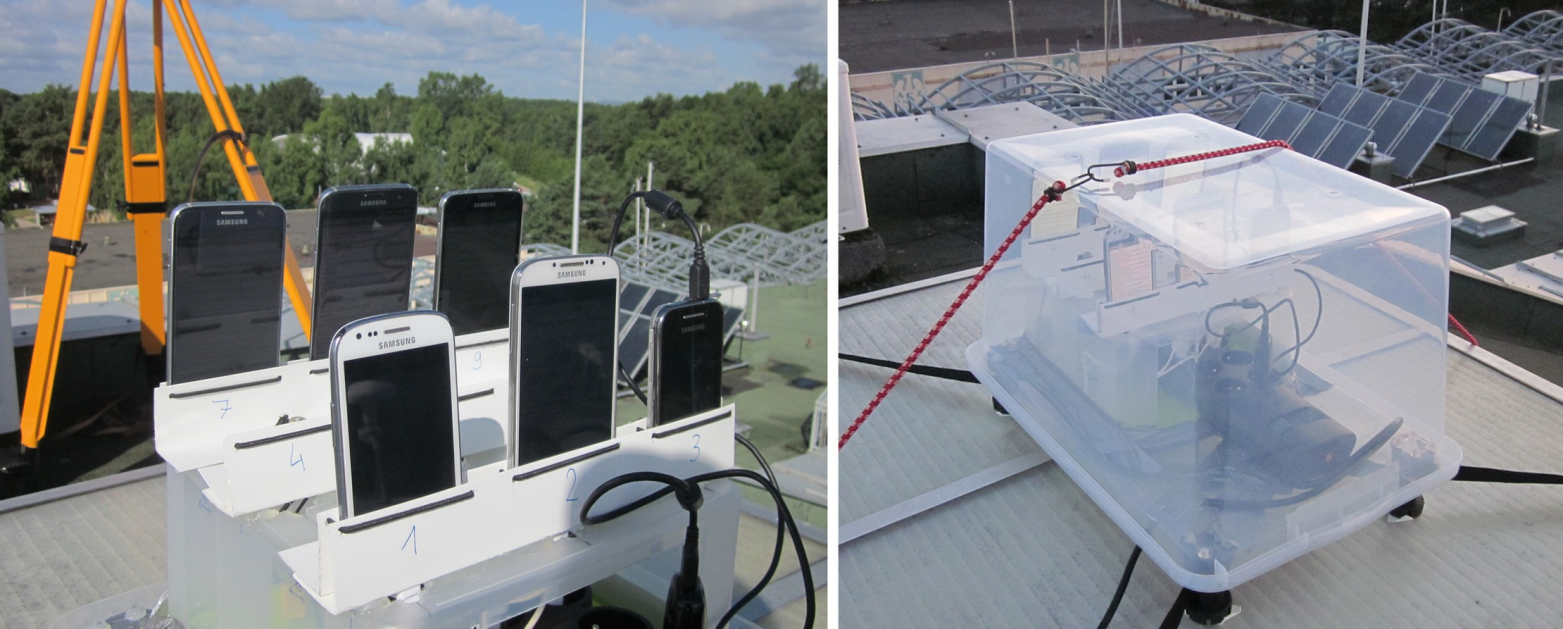
Measurement platform with attached mobile phones (left) and a plastic cover mounted (right).

As part of the research, two stationary measurement campaigns were held in 24-hour and 12-hour time spans respectively. The top roof of the National Sailing Centre in Gdansk (Poland) was chosen as the measurement site as location free from any terrain obstacles. A specially designed platform with vertically placed mobile phones was mounted on the roof (Fig 2). In case of unfavourable weather conditions (high humidity, drizzle, rain, etc.), a plastic cover tightly connected to the base could have been mounted on the platform to secure the devices (Fig 2). Inside the box a power strip with the connected phone chargers was placed.

The position coordinates determination by a smartphone receiver is substantially influenced by the device’s software [30]. Hence, it has been decided (like with [31]) to analyse only National Marine Electronics Association (NMEA) GGA messages recorded during measurement. Each smartphone used application developed by ppillaii [32] as a free NMEA GPS logger to register and record three-dimensional position coordinates and additional navigational parameters. All active applications (except for the NMEA logger), connection options (Wi-Fi, Bluetooth, mobile data) and power saving mode were disabled on each phone. The SIM cards were also removed. Only the location service remained enabled.

Before commencing stationary measurements by smartphones on the platform, it was necessary to accurately determine the reference (true) coordinates of mobile phones GNSS receivers. The centimetre accuracy of the determined coordinates was achieved by using satellite and classical geodetic measurement techniques. The real time kinematic (RTK) and real time network (RTN) solutions were not used. Instead, static satellite measurements of state-of-the-art geodetic GNSS receivers were used to establish a control network. In the next stage the tachymetry was used to determine coordinates of the antennas of the tested smartphones. The location of the four points of the control network was chosen during field reconnaissance. Steel bolts were used for permanent stabilisation.

**Fig 3:**
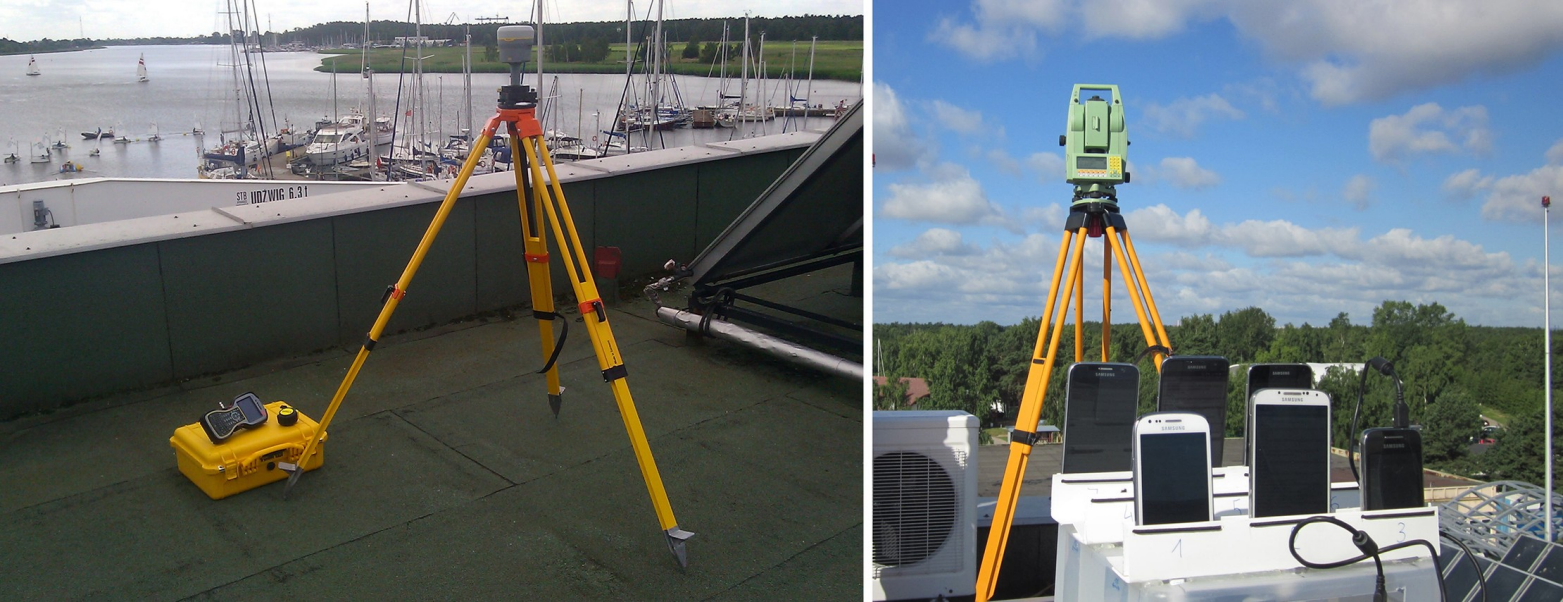
Establishment of the control network points using static satellite measurements (left) and determination of the position coordinates of the measurement station by the tachymetric method (right).

In the first phase of the survey, two GNSS Trimble R10 geodetic receivers were used to record raw satellite observations (Fig 3) during two 45-minute static sessions. Synchronous satellite observations of GPS and GLONASS systems were obtained from the reference stations of the TPI NETpro commercial active geodetic network located in Braniewo (68 km), Elblag (46 km), Gdansk (13 km), Starogard Gdanski (48 km) and Wejherowo (44 km). During post-processing precise ephemeris data in a ‘*.sp3’ format provided by GFZ German Research Centre for Geosciences was used. Satellite positions in the files were given with a 5-minute interval, which significantly increased accuracy of the determined GNSS vectors. All calculations were performed in the Trimble Business Center software. Estimated coordinates of the control network points were characterised by maximal errors of 0.010 m (horizontally) and 0.017 m (vertically). The resulting ellipsoidal coordinates were then transformed into the PL-2000 coordinate system (Polish modification of the Gauss-Kruger projection of the ellipsoid to the plane).

The second stage of the measurement work was determination of the position of the phones with the classic electronic distance measurements (EDM) using Leica TPS 1103 total station. The instrument position was calculated by the method of space resection, which requires the recording of directions and distances to points with known coordinates (Fig 3). Based on the set of four points of the control network, the station coordinates were determined with errors not exceeding 0.01 m. Known position and orientation of the instrument enabled the measurement of the positions of the phones and determination of the coordinates.

After determining the receivers phones coordinates the NMEA GPS application and the location service were activated, the “NMEA Raw Data” and “Save” options (for recording measurements on the microSD memory card) were selected. The first measurement campaign (24 hours) started at 13.15 (UTC + 2:00) on July 17 2017 and ended at 13.15 the next day. The second verification campaign (12 hours) started at 9.45 and ended at 21.45 on August 24, 2017. At night, the plastic cover was attached due to high humidity in the area. During the observations acquisition a number of problems was encountered:

- approximately 2–3 hours after the measurements started the applications in Samsung Galaxy S3 Mini and Y smartphones were suddenly shutting down. Consequently, it was decided to systematically record the information at 1.5-hour intervals (for Samsung S3 Mini and Y phones) and at 3-hour intervals for other phones, without removing them from the measurement platform;
- while recording data the NMEA GPS application occasionally crashed on some devices;
- at the beginning of the second measurement session, the Samsung Galaxy S7 application shutdown was observed, and a message about possible overheating was displayed. That was probably the result of a temporary plastic cover mounting due to the expected rain (despite the sunny summer day).

All the unexpected events resulted in the loss of unsaved data. Fortunately, these periods were relatively short because of the regular monitoring of a measurement platform status. After the measurements were completed, registered files were transferred to the computer and the obtained data were processed.

### Data processing

**Fig 4:**
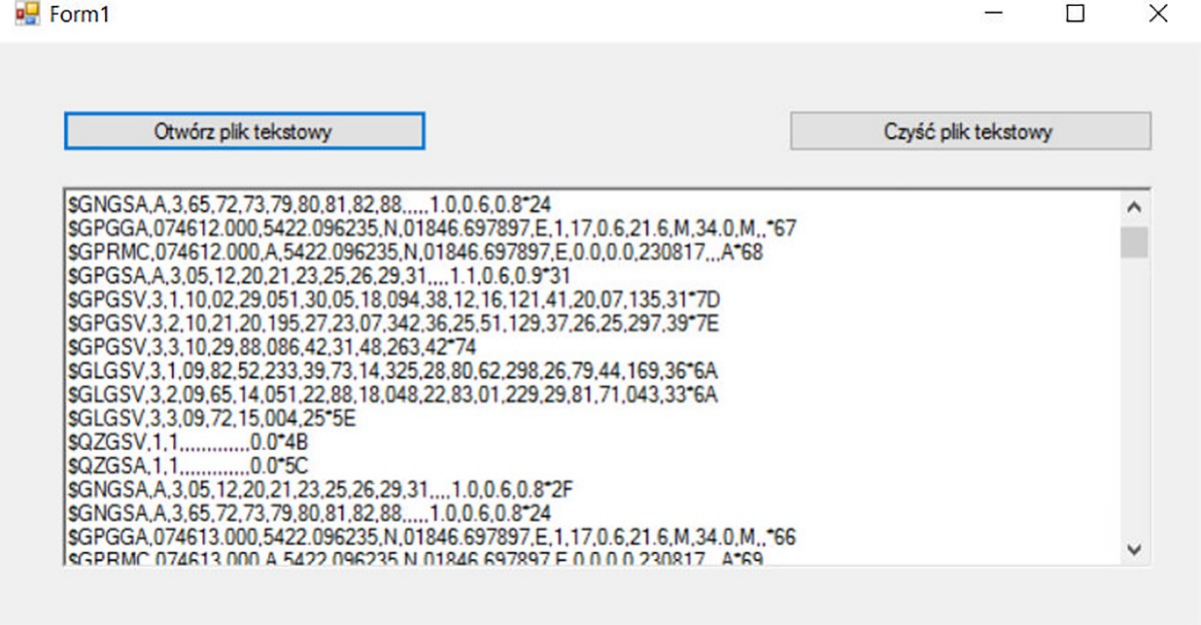
Software interface for deleting erroneously recorded NMEA-0183 standard data.

The recorded measurement data were merged from multiple minor files and saved in one observation file for each smartphone. The files were subsequently loaded to the proprietary software (in C#) for deleting erroneously recorded NMEA-0183 messages, quite common in GNSS receivers measurements datasets (Fig 4). It was used to calculate a checksum using the XOR operation on all characters of the line (except the leading dollar sign) to the checksum itself (without the checksum and the asterisk sign). The software compared a calculated checksum using an exclusive disjunction with the checksum value stored in the hexadecimal system at the end of each line. If checksum values were not consistent, the software deleted the selected line.

**Fig 5:**
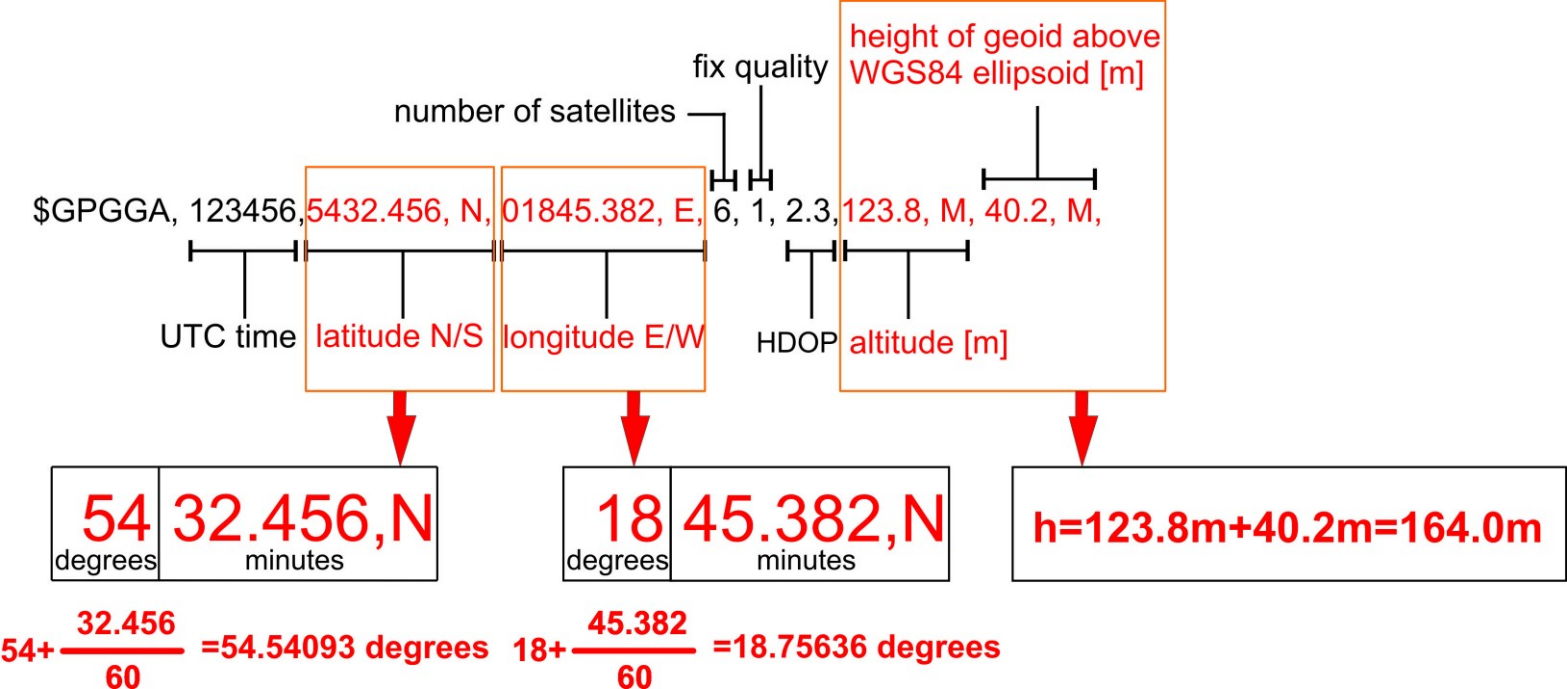
Description of the NMEA GGA message.

Upon completion of the software calculation, three files containing erroneously recorded NMEA-0183 messages (1), NMEA GGA messages (2) and other messages (3) were received. Only GGA messages were used in the research. In the context of further analyses, it is important to explain in details the format of the latitude and longitude coordinates fields registered in the form of full degrees and minutes within one string. It needed to be converted to full degrees according to the example presented in the Fig 5.

The full-degree coordinates format was needed for further conversion of angular latitude and longitude into metric Cartesian coordinates. The second important operation was the normal height calculation based on the altitude above mean sea level (GGA message) and the height of geoid above World Geodetic System 1984 (WGS84) ellipsoid obtained by interpolation of the EGM2008 geoid model [33]. Formulas in [34] were used to determine the normal heights.

h=H+N=H*+ζ (1)

where:

*h* - geodetic height (height relative to the ellipsoid),

*H* - orthometric height (height relative to the geoid),

*N* - geoid undulation,

H* - normal height,

*ζ* - height anomaly.

Determining errors of individual measurements (expressed in the differences of angular horizontal coordinates of latitude and longitude) due to the changing length of parallels arcs requires introducing a projection to present them as linear values in meters. To accomplish that the angular coordinates of the WGS84 ellipsoid (with parameters: a = 6378137.000 m, b = 6356752.314 m) [34] were projected onto a surface using the Gauss-Kruger projection commonly used in geodesy [35]. As a result of the calculations, the plane coordinates (x, y) were obtained, where x denotes distance in meters of the point from the equator calculated along the meridian arc (on the WGS84 ellipsoid), and y denotes distance in meters from the central meridian of several degrees wide zone, which is arbitrarily set. A minus sign in y coordinate means that the point is located west of the meridian, whereas a plus sign corresponds with a position to the east of the meridian. In order to avoid negative values of coordinates on the y axis, a constant value of, for instance, 500 000 m (for the three-degree longitude zone in the Polish 2000 system) is added to the coordinate. The entire process of converting the angular ellipsoidal coordinates into Cartesian coordinates was based on the formulas presented in [36].

After converting ellipsoidal coordinates of points to plane coordinates and determining normal heights, the results were statistically calculated. For this purpose, the commonly used navigation method of measuring the accuracy of positioning was used, which is summarised in Table 2 [37–39].

**Table 2:** Selected position accuracy measures.

Accuracy measure	Dimension	Probability	Definition
**RMS**	1D	68%	The root mean squared error calculated for φ, λ or h.
**DRMS**	2D	63–68%	The distance root mean squared error calculated for φ, λ, (h).
	3D		
**2DRMS**	2D	95–98%	Twice the DRMS.
	3D		
**CEP**	2D	50%	The radius of circle centred at the true position, containing the position estimate with probability of 50%.
**SEP**	3D	50%	The radius of sphere centred at the true position, containing the position estimate with probability of 50%.
**R68**	2D	68%	The radius of circle (sphere) centred at the true position, containing the position estimate with probability of 68%.
	3D		
**R95**	2D	95%	The radius of circle (sphere) centred at the true position, containing the position estimate with probability of 95%.
	3D		
where: σ_φ_−standard deviation of geodetic (geographic) latitude; σ_λ_−standard deviation of geodetic (geographic) longitude; σ_h_−standard deviation of ellipsoidal height.			

Horizontal coordinates of the latitude and longitude (φ, λ) and orthometric height (H) were recorded in NMEA GGA format files. On their basis, the RMS values, used in further formulas in Table 2, were calculated. RMS _φ_ and RMS _λ_ parameters are initially expressed in angular units (degrees). In order to determine the linear values of positioning errors, the values needed to be related to the one-degree arcs lengths of meridian and parallel in the measurement location (Gdansk). The length of the meridian arc is calculated using the following formula:

$$s_{M}=\int_{\varphi_{1}}^{\varphi_{2}}M\left(\varphi \right)d\varphi =\int_{\varphi_{1}}^{\varphi_{2}}\frac{a\cdot \left(1-e^{2}\right)}{\sqrt{{\left(1-e^{2}\sin^{2}\left(\varphi \right)\right)}^{3}}}d\varphi$$ (2)

where:

*s*_*M*_ - meridian arc length of the ellipsoid of revolution,

*M*(*φ*) - the radius of curvature in the meridian plane at latitude φ,

*φ*_1_,*φ*_2_ - latitudes of points determining the meridian arc,

*a*- major semi-axis of the ellipsoid of revolution length,

*e* - ellipsoid of revolution eccentricity.

In order to determine the arc length of the parallel, it is necessary to calculate its radius length using the formula:

$$r=N\left(\varphi \right)\cos\left(\varphi \right)=\frac{a}{\sqrt{1-e^{2}\sin^{2}\left(\varphi \right)}}\cos\left(\varphi \right)$$ (3)

where:

*N*(*φ*) - the radius of curvature in the prime vertical plane at latitude φ.

The next step is to calculate the length of the parallel circle arc. According to the angle definition, this length was obtained using the formula:

$$s_{P}=r\cdot \overset{⌢}{\alpha }$$ (4)

where:

*s*_*P*_ - parallel arc length,

$\overset{⌢}{\alpha }$ - angle in the radial measure.

In case of the WGS84 ellipsoid of revolution used as the reference, the one-degree arcs lengths of meridian (s _M_) and parallel (s _P_) in Gdansk were 111 311.842 m and 64 995.633 m, respectively. According to them, the linear values of RMS _φ_ and RMS _λ_ were calculated.

## Results

The assumption of the study was to conduct the measurement continuously for a period of 24 hours with a one-second interval in the same conditions. As a result, a representative synchronous sample of statistical data that describe the smartphones positioning process was obtained. The collected data allowed to carry out a reliable comparative accuracy analysis. By determining the coordinates of the GNSS receivers of the phones, the previously described accuracy measures of positioning could be related to actual values (predictable accuracy). Due to the limited volume of the article, statistics of errors in the repeatable position were deliberately omitted. The applied approach can be found in the geodesy and navigation literature. For the evaluation of several adopted measures of accuracy values a Mathcad sheet was created (Table 3). Due to the achieved low accuracy of the positioning of Samsung Galaxy S6 and S7 phones additional verification session was conducted (Table 4).

**Table 3:** Statistics of position errors of Samsung Galaxy phones calculated for the 24-hour measurement campaign.

Statistics of position error	Y	S3 Mini	S4	S5	S6	S7
**Number of measurements**	73 699	71 438	86 290	86 346	86 371	86 355
**RMS (φ)**	2.47 m	2.46 m	0.70 m	0.65 m	5.87 m	3.93 m
**RMS (λ)**	1.33 m	2.34 m	0.74 m	0.80 m	3.52 m	2.11 m
**RMS (h)**	4.36 m	1.11 m	1.74 m	2.60 m	11.11 m	6.04 m
**DRMS (2D)**	2.81 m	3.39 m	1.02 m	1.03 m	6.84 m	4.46 m
**2DRMS (2D)**	5.61 m	6.79 m	2.04 m	2.06 m	13.69 m	8.93 m
**DRMS (3D)**	5.18 m	3.57 m	2.01 m	2.79 m	13.05 m	7.51 m
**CEP (2D)**	1.60 m	3.76 m	0.88 m	0.87 m	4.31 m	3.24 m
**R68 (2D)**	3.71 m	3.76 m	1.10 m	0.97 m	5.92 m	4.37 m
**R95 (2D)**	4.93 m	3.76 m	1.65 m	1.76 m	12.64 m	8.39 m
**SEP (3D)**	4.24 m	3.84 m	1.78 m	3.19 m	12.22 m	6.68 m
**R68 (3D)**	5.03 m	3.84 m	1.93 m	3.22 m	14.58 m	8.21 m
**R95 (3D)**	9.13 m	3.84 m	3.53 m	3.74 m	18.96 m	12.26 m

**Table 4:** Statistics of position errors of Samsung Galaxy phones calculated for the 12-hour measurement campaign.

Statistics of position error	Y	S3 Mini	S4	S5	S6	S7
**Number of measurements**	43 013	37 771	43 159	43 181	43 185	31 835
**RMS (φ)**	1.56 m	0.96 m	1.02 m	1.63 m	7.48 m	5.74 m
**RMS (λ)**	1.75 m	1.40 m	0.88 m	1.89 m	2.70 m	2.21 m
**RMS (h)**	4.71 m	2.71 m	0.97 m	2.83 m	9.99 m	9.73 m
**DRMS (2D)**	2.34 m	1.70 m	1.35 m	2.49 m	7.95 m	6.15 m
**2DRMS (2D)**	4.68 m	3.39 m	2.70 m	4.98 m	15.90 m	12.29 m
**DRMS (3D)**	5.26 m	3.20 m	1.66 m	3.77 m	12.77 m	11.51 m
**CEP (2D)**	2.14 m	1.48 m	1.01 m	1.78 m	3.82 m	3.84 m
**R68 (2D)**	2.45 m	1.48 m	1.38 m	2.10 m	5.36 m	5.45 m
**R95 (2D)**	4.42 m	2.79 m	1.86 m	4.97 m	17.88 m	12.94 m
**SEP (3D)**	3.76 m	2.58 m	1.52 m	2.99 m	10.86 m	10.34 m
**R68 (3D)**	4.97 m	2.58 m	1.68 m	4.38 m	12.56 m	11.83 m
**R95 (3D)**	9.92 m	6.05 m	2.24 m	5.89 m	22.74 m	18.57 m

**Fig 6:**
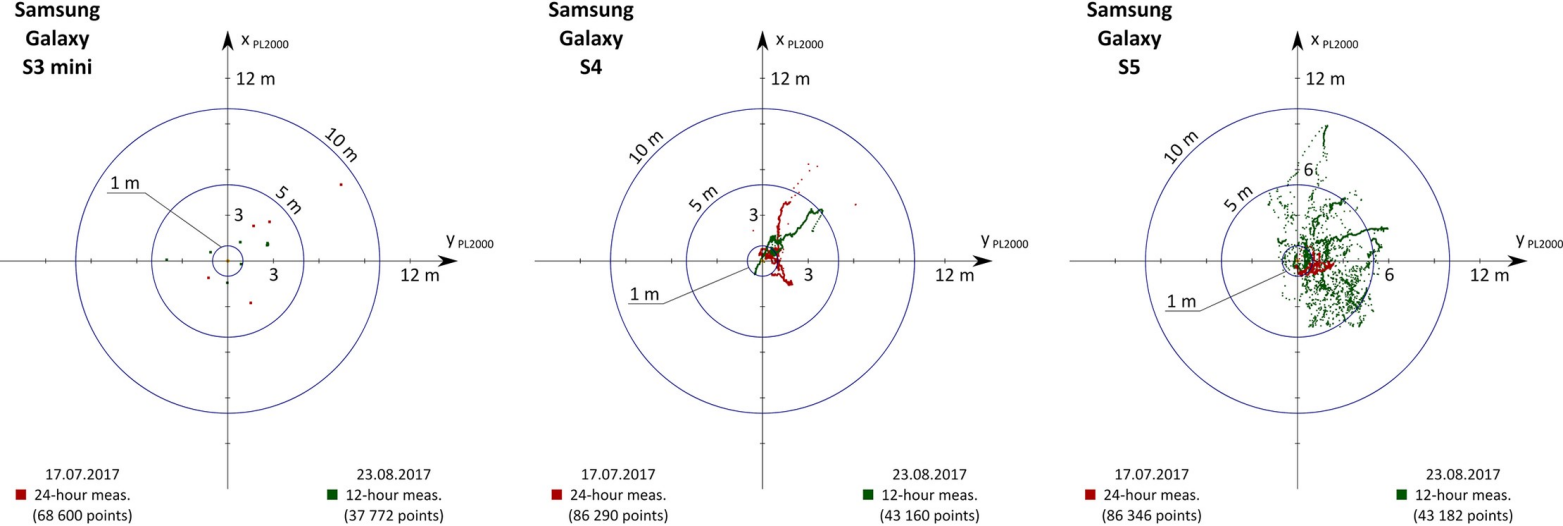
Two measurement campaigns results of Samsung S3 mini, S4 and S5 smartphones.

Based on the results presented in Table 3, the smallest error was obtained in the Samsung Galaxy S4 smartphone, which achieved 2D results: 1.10 m (R68) and 1.65 m (R95), and in the 3D: 1.93 m (R68) and 3.53 m (R95). Similar, but slightly lower accuracy characteristics are those of a Samsung Galaxy S5 (differences between various measures are usually from a dozen to several dozen cm)—Fig 6. On the Samsung Galaxy S3 Mini phone, most of the accuracy measures are within the range of 3–4 m (both horizontal and vertical). However, during processing of the measurement data it was noted that the phone continuously recorded the same position during individual 1.5-hour measurement sessions (the coordinates changed when the NMEA GGA file saving option was activated). A value decrease in the horizontal position determination errors (RMS (φ) lower by 61% and RMS (λ) lower by 40%) was noted. In the case of RMS (h), the error increased by 144% to 2.71 m in the second measurement campaign. The above accuracy measures cannot be regarded as reliable for a S3 Mini, however, due to the small statistical sample. Particularly noteworthy is the diametrically different measurements precision of the S5 smartphone. In the first measurement campaign, low values of accuracy parameters of 1.03 m (DRMS (2D)) and 2.79 m (DRMS (3D)) were obtained. The second measurement campaign showed a significant increase in the value of DRMS (2D) by 142% from the value of 1.03 m to 2.49 m, which can be seen in Fig 6. It should be noted that the RMS (h) error has practically not changed (increase by 9% from a value of 2.6 m to 2.83 m). Accuracy measures calculated for the S4 smartphone do not differ between the two measurement campaigns by more than 50% (Tables 3 and 4).

**Fig 7:**
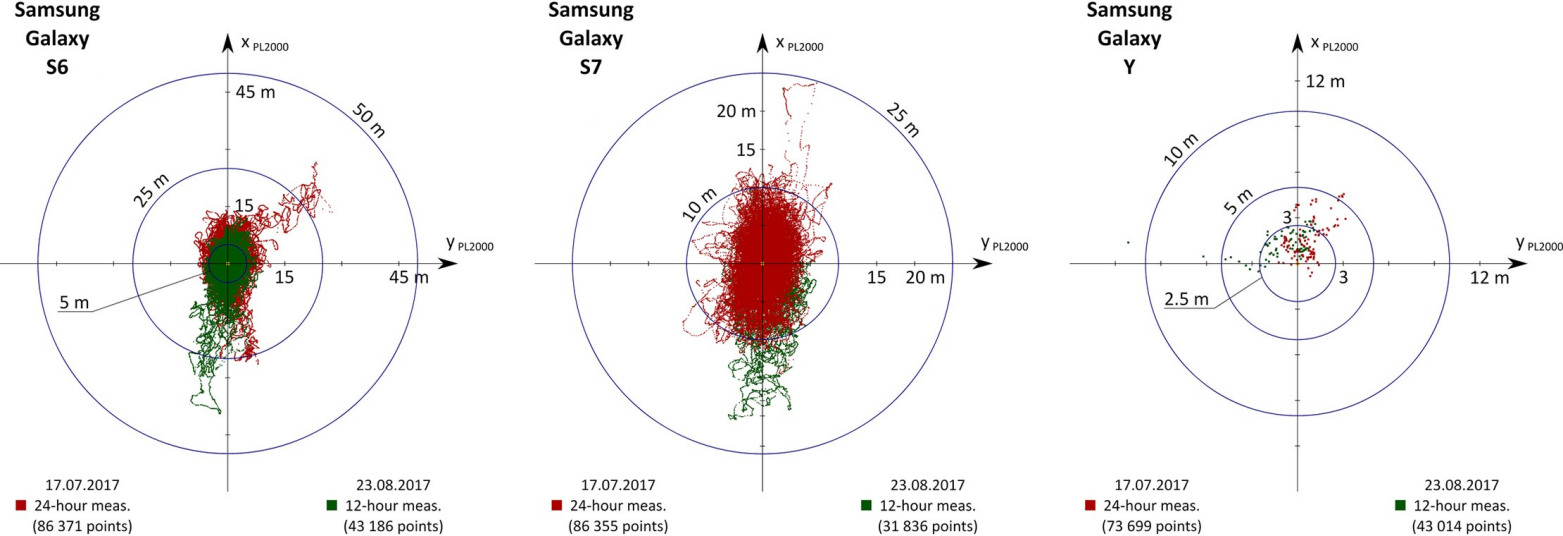
Two measurement campaigns results of Samsung S6 mini, S7 and Y smartphones.

The results of position determination of the other three remaining smartphone models in two measurement campaigns are presented in Fig 7. The obtained accuracy measures in most cases do not differ by more than 40%. The smallest differences were noted in the S6 model. In the first 24-hour campaign, the Samsung Galaxy S6 obtained errors of 6.84 m (DRMS (2D)) and 13.05 m (DRMS (3D)), which is a significant deterioration of positioning accuracy compared to older models (Table 3). The next generation of the Galaxy series smartphone—S7 in the tests obtained lower values of accuracy characteristics: 4.46 m (DRMS (2D)) and 7.51 m (DRMS (3D)) in the first measurement campaign. However, an increase in the RMS (φ) (by 46%) and RMS (h) errors (by 61%) in the second campaign resulted in higher values of DRMS (2D) and DRMS (3D) of 6.15 m and 11.51 m (Table 4). The S6 and S7 models turned out to be the least precise devices in the whole set. Almost all their measurements, both in 2D and 3D, vary from almost 10 to even a dozen or so meters. The next smartphone, which supported only GPS satellites, (Samsung Galaxy Y) fulfils the accuracy requirements of [40], and the previously quoted position errors are, respectively: 3.71 m (R68) and 4.93 m (R95) in the 2D plane, as well as 5.03 m (R68) and 9.13 m (R95) in the 3D plane. In addition, the Samsung Galaxy Y recorded periodically the same position, but definitely less often than the S3 Mini (Figs 6 and 7).

The study also analyses the random variable distribution which is the error of a single measurement in terms of typical statistics used in navigation [41]. According to the Central Limit Theorem it can be assumed that the random variables distribution with a large number of variable values follow to a normal distribution [42]. Given the number of coordinate errors in the north and east directions (x and y) considered in the research, their normal distribution can be assumed. The linear position determination errors are defined by the Pythagorean theorem and the calculation of the hypotenuse length. This formula corresponds to Euclidean norm of two independent random variables. According to the definition, the probability distribution of the resulting variable is Rayleigh distribution. Hence, it can be expected that the empirical probability distribution of the accuracy indicators determined in this work will have a distribution similar to the Rayleigh distribution. The statistical distribution of position errors can be presented by Rayleigh's distribution, as probability density function ƒ(x; σ) and distribution function F(x):

$$f\left(x;\sigma \right)=\frac{x}{\sigma^{2}}e^{\frac{‐x^{2}}{2\sigma^{2}}},\;F\left(x\right)=1‐e^{\frac{‐x^{2}}{2\sigma^{2}}}$$ (5, 6)

for *x*∈[0,∞), where the scale parameter σ is defined as:

$$\sigma =\sqrt{\frac{1}{2N}\sum_{i=1}^{N}x_{i}{}^{2}}$$ (7)

where:

N–number of measurements.

**Fig 8:**
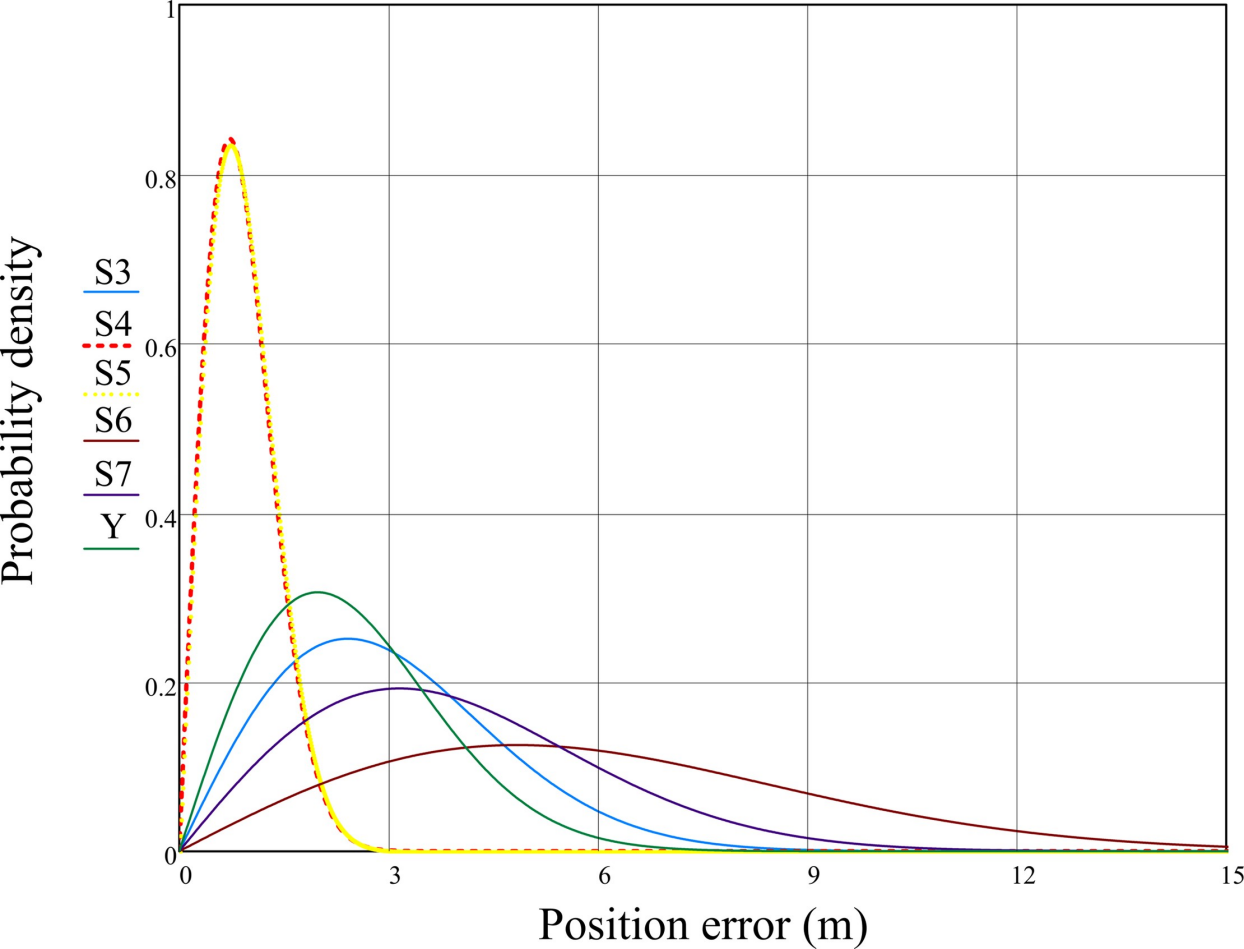
2D Rayleigh probability density function of Samsung Galaxy phones calculated for the 24-hour measurement campaign.

The probability density functions (PDFs) present the probability of a continuous random variable’s (in this case a position error) taking a value from a particular given range. It is worth noting that statistical tests carried out in the EasyFit 5.5 Professional software confirm that the position error empirical probability distribution is similar to the Rayleigh distribution (5). The functions have the property that its integral over the entire domain (area under the function graph) is equal to one. Depending on the parameters of the expected value and the standard deviation, the PDF functions graph shape differs. The expected value in the case of repeatable accuracy analysis is the arithmetic mean. In the presented analysis of the predicted accuracy it is the known true value (the reference coordinates of smartphones determined using precise surveying instruments). The standard deviation of a variable can be interpreted in the general Rayleigh distribution as a scale parameter [42–43]. Hence, high accuracy GNSS receivers should have PDF functions fast monotonically increasing and close to the vertical axis graphs. The GNSS receivers of the S4 and S5 smartphones determined the position with a very similar and relatively small error in relation to the other tested models. Furthermore, the graph of their probability density function shows that they obtained small values of standard error deviations. Increasing flattening and increasing values of the functions graphs maximum arguments prove the lower the accuracy and the precision of subsequent models (Y, S3 Mini, S7 and S6) (Fig 8).

**Fig 9:**
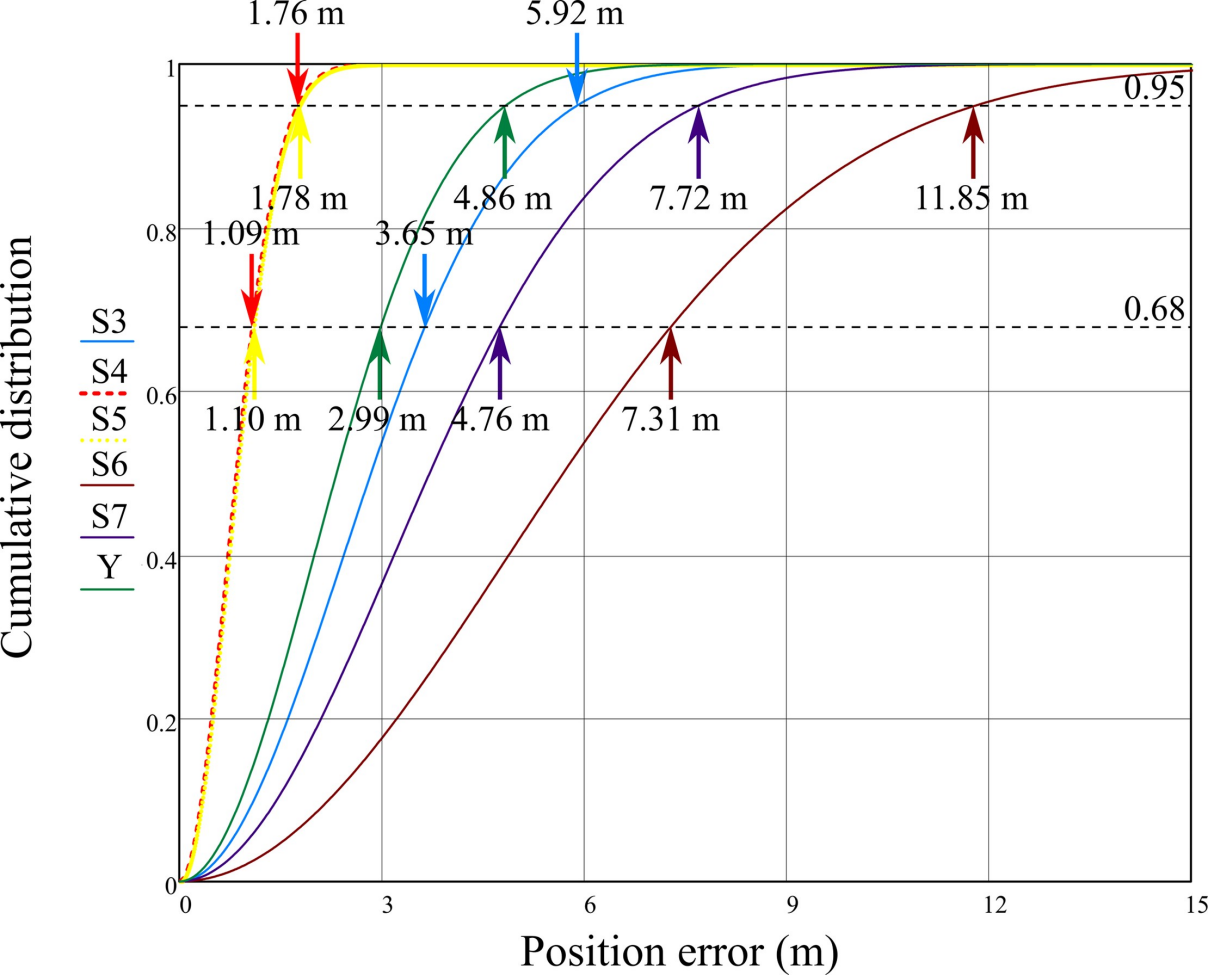
2D Rayleigh distribution function of Samsung Galaxy phones calculated for the 24-hour measurement campaign.

Cumulative distribution functions (CDFs) represent the probability that the random variable will take a value less than or equal to a given argument (Fig 9). In this case the definition should be read as the probability (vertical axis) of a position determination error equal to or lower than the given threshold value (horizontal axis). The fast monotonically increasing function graph shows the high accuracy of the measuring device. The CDF functions (6) are strictly dependent on the PDF functions (5). Additionally, in Fig 9 lines of the probabilities of 68% (0.68) and 95% (0.95) were added with corresponding values of the tested smartphones CDF functions determined by the Rayleigh distribution quantile function formula that can be derived from (6):

$$Q=\sigma \sqrt{-2\ln\left(1-p\right)}$$ (8)

where:

*Q* - random variable value (positioning error),

*p* - probability threshold value.

Similar results of S4 and S5 smartphones confirm the two model’s relatively high accuracy in comparison to other devices (steep chart of the function of the distribution function). The probability that the position assigned with them will be burdened with an error greater than 3 m is almost certain (99.9%). Similarly, in the case of the S6 model, the probability is only 36.4%.

## Discussion

Table 5 shows the global scientific research carried out over the last few years, during which the accuracy of static (stationary method) position determination in the group of smartphones and sport-recreational receivers was assessed. It is worth adding that there is much more research on the assessment of kinematic position determination accuracy [43–47], [31] than stationary described in this research.

**Table 5:** Assessment of the accuracy of determining the position of smartphones and sport-recreational receivers using static measurements.

Author & purpose of research	Type of measurement	Accuracy measure	Number of points recorded	Mobile application, smartphone & obtained accuracy or receiver & obtained accuracy
**Smartphone app performance during various weather conditions [48]**	Static (known position)	Avg horizontal error, StDev, median, max and min. error	12 200	U-Center GPS app; Samsung Galaxy Mini: 5.11–5.4 m (avg horizontal error), 3.37–4.74 m (StDev), 3.83–4.17 m (median), 21.38–46.13 m (max error), 0.22–2.09 m (min. error)
**Determining the validity of portable GPS receivers [49]**	Static (geodetic sites are determined with an accuracy of 0.10–0.15 m)	CEP, mean error	N/A	FRWD B100: 6.8 m (CEP), 29.0 ± 93.7 m (mean error); Garmin Forerunner 205: 5.7 m (CEP), 13.9 ± 21.5 m (mean error); Garmin Foretrex 201: 5.5 m (CEP), 14.6 ± 26.8 m (mean error); GlobalSat TR-203: 6.3 m (CEP), 58.8 ± 393.2 m (mean error); i-gotU GT-600 Logger: 10.8 m (CEP), 19.6 ± 30.9 m (mean error); Qstarz BT-Q1000XT: 5.0 m (CEP), 12.1 ± 19.6 m (mean error); StarsNav BTS-110: 7.3 m (CEP), 12.3 ± 15.6 m (mean error)
**Assessing the accuracy of various GNSS receivers [50]**	Static (geodetic point is determined with an accuracy of < 1 cm)	2DRMS	9 000	Garmin Edge 205: 4.02 m; Garmin Forerunner 305: 7.06 m; Pentagram P3106: 2.84 m; Qstarz BT-Q1000: 2.18 m; Wintec WBT-100: 2.97 m

It can be seen in Table 5 that researchers use different methods to assess the accuracy of measurements, while the basic and most commonly used in geodetic and navigational literature accuracy measures of position coordinates are: RMS, DRMS, 2DRMS, CEP, SEP, R68 and R95 (described in data processing section). The positioning accuracy of Samsung Galaxy series smartphones GNSS receivers exceeded the accuracy provided by GPS receivers tested in [49]. The CEP error values of both the first and the second smartphone measurement campaigns are lower than in the cited publication. The tested GPS receivers did not obtain a value lower than 5.0 m (Qstarz BT-Q1000XT). The worst position in terms of accuracy was obtained by i-gotU GT-600 Logger (10.8 m). Bearing in mind the year of publication (2013) and only one GNSS system used (GPS), it should be noted that Samsung Galaxy Y (present on the market since 2011), S3 Mini at the time (since 2012) and S4 (since 2013) have obtained much better accuracy parameters. Therefore, using them as sports route loggers seems justified due to the higher accuracy of positioning.

[50] describes the determination of the reference coordinates values with the same accuracy as obtained in the following research (< 1 cm). The accuracy criterion used for the parameterisation of sports GNSS receivers was 2DRMS—a measure commonly used in surveying and navigation. As a result of the obtained values comparison, it should be noted that only two smartphones (S4 and S5) and in the first campaign only, achieved better positioning accuracy than the tested sports receivers. Qstarz BT-Q1000 with the accuracy of 2.18 m proved to be the most accurate GNSS receiver. The remaining models also obtained good results (2.18 m, 2.97 m, 4.02 m and 7.06 m) compared to Samsung Galaxy smartphones, whose 2DRMS values usually oscillated between 5 and 6 m (Y, S3 Mini, S5) with several values exceeding 10 m (S6 and S7). The accuracy of sports receivers described in [50] proves their higher accuracy and precision in relation to Samsung Galaxy smartphones.

## Conclusions

While addressing the issue of accuracy of GNSS receivers implemented in smartphones, the authors realised that these are very specific measurement devices. The final effect of displaying the desired information (a pair of coordinates, speed, direction, position on the electronic map) is affected by a number of factors that can be assigned to two groups: (1) those who are fully or partially user-dependent (software update and selection, choice of specific software options, firmware update in the device, etc.); and (2) factors that the user cannot alter (the quality of components installed in the receiver, built-in systems software, GNSS system errors, etc.). The popularity of smartphones and their use in navigational applications is widespread in public, but just few nonprofessional users are aware of the complexity of the underlying process, based on the knowledge and experience of hardware and software manufacturers. It would be helpful if smartphones manufacturers were to act like universal GNSS data loggers manufacturers (e.g. Qstarz, Holux) and published technical information on the achieved accuracy or on implemented GNSS chipsets. Unfortunately, smartphone specifications are missing such data. Hence, the major objective of this article has been to bridge the gap.

Determining the position using smartphones in most cases is used in dynamic applications (travelled route registration, road navigation, etc.). Arbitrary determination of kinematic accuracy is difficult due to the changing measurement conditions (speed, direction, obstacles presence, etc.). For this reason, long-term stationary measurements are carried out in favourable field conditions (no obstacles) to assess the accuracy of smartphones. Considering the ideal conditions for carrying out the measurement, it can be assumed that smartphones in everyday use in urban areas will achieve accuracy in the best case equal to that obtained in the study. The authors aimed to verify the hypothesis concerning satisfying the needs for better positioning accuracies by successive generations of mobile devices. Due to the popularity, availability and recognized position on the smartphone market, six models of the Samsung Galaxy series from 2011–2016 were used in the study (Y, S3 Mini, S4, S5, S6 and S7). The construction of a dedicated measurement platform enabled synchronous measurements under the same field conditions. To verify the obtained results of the 24 hour long measurement, the second independent campaign lasting 12 hours was carried out in the same place. The one-second measurement interval ensured obtaining a representative statistical sample. The collected measurement data were processed to determine the error values commonly used in surveying and navigation (RMS, DRMS, CEP and others). In addition, a statistical analysis of the probability distribution of a random variable of measurement error was performed.

Two of the tested phones obtained the 2DRMS errors in the first campaign that slightly exceeded 2 m (S4 and S5). The oldest models (Y and S3 Mini) obtained three times greater error values and two latest tested smartphones obtained a surprising result close to 14 m (S6) and 9 m (S7) accuracy. According to that, the second measurement campaign was performed to verify the original research. For most cases, the accuracy obtained by smartphones was confirmed. Only Samsung Galaxy S5 got significantly different accuracy parameters (e.g. 2DRMS larger by 142%). Errors increments exceeding 50% of the value obtained in the first campaign were also recorded in smartphones S3 Mini and S7. In other cases (Y, S4 and S6 models), the error differences did not exceed 50%, with a significant part not exceeding 25% of the original values. The oldest models of the Samsung Galaxy series (Y and S3 Mini) during the tests measured the position abruptly, often registering the same coordinates for a longer period of time. The significant low accuracy confirmation in two measuring campaigns was found in the case of the S6, which additionally confirmed the worst result in the smartphones group. Obtained results show that intuitive perception of newer Galaxy models as better in every possible operational aspect does not necessarily refer to the accuracy of positioning. It is difficult to understand considering that the operators of global navigation satellite/positioning systems continue to develop and improve them. It is also worth noting that the research was carried out at a measuring station without terrain obstacles—the conditions rarely encountered in everyday use of such receivers.

The limitation of the research is that only one model of each smartphone was tested in measurement campaigns. In future work more than one device per phone type will be validated to present the repeatability of the determined positioning accuracies.
